# What is a melody? On the relationship between pitch and brightness of timbre

**DOI:** 10.3389/fnsys.2013.00127

**Published:** 2014-01-17

**Authors:** Marion Cousineau, Samuele Carcagno, Laurent Demany, Daniel Pressnitzer

**Affiliations:** ^1^International Laboratory for Brain, Music and Sound Research (BRAMS), Department of Psychology, University of MontrealMontreal, QC, Canada; ^2^CNRS and Université de BordeauxBordeaux, France; ^3^Laboratoire des Systèmes Perceptifs, CNRS UMR 8248Paris, France; ^4^Département d’études cognitives, École normale supérieureParis, France

**Keywords:** pitch, timbre, brightness, melodies, sequences

## Abstract

Previous studies showed that the perceptual processing of sound sequences is more efficient when the sounds vary in pitch than when they vary in loudness. We show here that sequences of sounds varying in brightness of timbre are processed with the same efficiency as pitch sequences. The sounds used consisted of two simultaneous pure tones one octave apart, and the listeners’ task was to make same/different judgments on pairs of sequences varying in length (one, two, or four sounds). In one condition, brightness of timbre was varied within the sequences by changing the relative level of the two pure tones. In other conditions, pitch was varied by changing fundamental frequency, or loudness was varied by changing the overall level. In all conditions, only two possible sounds could be used in a given sequence, and these two sounds were equally discriminable. When sequence length increased from one to four, discrimination performance decreased substantially for loudness sequences, but to a smaller extent for brightness sequences and pitch sequences. In the latter two conditions, sequence length had a similar effect on performance. These results suggest that the processes dedicated to pitch and brightness analysis, when probed with a sequence-discrimination task, share unexpected similarities.

## Introduction

In many musical traditions, including Western tonal music, melodies have a key structural role (e.g., Dowling and Harwood, 1986). Providing a general but precise definition of a musical melody is somewhat difficult, but at the core, a melody can be thought of as a sequence of sounds organized along the pitch and rhythm dimensions. Importantly, timbre does not appear in this basic definition: a given melody remains the same even when it is sung on different words, or played with different instruments. This suggests that pitch and timbre have distinct roles in music. One may then wonder whether such a distinction stems from basic perceptual characteristics or whether it is purely coincidental.

A first thing to note is that although pitch and timbre generally have a different status in music, there are exceptions. Indian tabla music, for instance, is organized around timbre contrasts (Patel, 2008, p. 36). In Western musical history, Schoenberg (1911) famously advocated the use of timbre as a structural element for melodic writing, coining the term *Klangfarbenmelodie*. He went as far as to question the traditional distinction between pitch and timbre:

*“I cannot admit without reservations the distinction between timbre* (*tone color,* Klangfarben)* and pitch* (Klanghöhe), *as it is usually expressed. [….] Pitch is nothing but timbre measured in one direction.”* (Schoenberg, 1911, p. 471).

Striking examples of Klangfarbenmelodie are found in, e.g., Webern’s Opus 10. More recently, contemporary composers such as the proponents of the “spectral music” style have also used timbre as a central musical device (Grisey and Fineberg, 2000). And it could be argued that a lot of the current “electronica” genre relies on manipulations of timbre. Thus, at least some musical idioms use timbre as a form-bearing entity.

In the psychophysical experiment reported here, we compare the perception of sequences of sounds that differ along the pitch dimension to the perception of sequences of sounds that differ in timbre. The paradigm has been originally used to compare pitch and loudness sequence processing (Cousineau et al., 2009). The core idea is to construct sequences made out of elements that are all equally discriminable, regardless of the dimension of interest. The experiment starts with an adjustment phase: discriminability is measured, in turn, along the different dimensions to be compared (e.g., fundamental frequency and sound pressure level (SPL) for pitch and loudness). For each listener and dimension, a step size is derived, targeting equal discriminability. In the main part of the experiment, random binary sequences are constructed, which are simply a succession of sounds that can take one of two values on the dimension of interest. The two values differ by the equal-discriminability step. Listeners have to perform a same/different task on pairs of sequences, with varying numbers of elements in the sequences. The aim of the paradigm is to provide meaningful comparisons for sequence processing along different dimensions, factoring out the influence of perceptual distance between the elements constituting the sequences.

Using this technique, we found previously that listeners performed better for pitch sequences than for loudness sequences (Cousineau et al., 2009). We also found that the pitch-sequence advantage was conditional on the availability of peripherally-resolved harmonics (Cousineau et al., 2009, 2010a,b). This led to the hypothesis that pitch sequences could benefit from specific perceptual processes compared to, e.g., loudness sequences. As most musical sounds contain resolved harmonics, the benefit ought to apply to musical melodies.

Here, we extend the sequence processing paradigm to timbre. Because timbre is a multidimensional attribute (e.g., Patil et al., 2012; Elliott et al., 2013), a single dimension was selected to construct univariate sequences. We chose the dimension termed “brightness”, which is related to the mean of the distribution of spectral energy in a sound (Grey, 1977; McAdams et al., 1995). As an illustrative example, playing the violin with the bow close to the bridge (*sul ponticello*) will produce many harmonics and a bright sound, whereas playing the same note with the bow close to the fingerboard (*sul tasto*) will produce relatively fewer harmonics and a sound that is less bright. In the present experiment, we used a much-simplified acoustic manipulation that allows for the variation of brightness. Our sound stimuli always consisted of two simultaneous pure tones one octave apart. In one of the three experimental conditions, we constructed brightness sequences by varying the level balance of the two tones. In a second condition, we constructed pitch sequences by varying the frequency of both tones while maintaining their octave relationship. In the third condition, we constructed loudness sequences by varying overall level. From previous results (Cousineau et al., 2009), we expected an advantage for pitch sequences compared to loudness sequences. The main question concerned the brightness sequences: we wished to determine whether they would be processed like pitch sequences or rather like loudness sequences.

Relevant to this question are psychoacoustic investigations of perceptual interactions between pitch and brightness: if pitch and brightness share common processing resources, they should interact strongly. Evidence is mixed on this issue. On one side of the argument, there are many studies showing a relative independence of pitch and brightness processing. Demany and Semal (1993) measured the detection of small shifts in pitch (fundamental frequency) combined with small shifts in brightness (spectral centroid); they found that the relative directions of the shifts (e.g., higher pitch combined with brighter timbre vs. lower pitch combined with brighter timbre) had no effect on detection performance. This suggests that the two dimensions are processed independently, at least for small shifts. Short-term memory for pitch and for timbre have also been found to be largely independent. When listeners have to compare pairs of target tones separated by intervening tones, pitch judgments on the target tones are immune to interference from the brightness of the intervening tones (Semal and Demany, 1991); vice versa, the pitch of the intervening tones does not affect timbre judgments on the target tones (Starr and Pitt, 1997).

On the other side of the argument, there is also evidence for interactions between pitch and brightness processing. Melara and Marks (1990) investigated the speeded classification of sounds on the pitch or brightness dimension, while the other dimension was either held constant, varied congruously, or varied incongruously (Garner interference task: Garner, 1974). Congruity effects were observed: for instance, a high pitch was classified faster when accompanied by high brightness, compared to high pitch and low brightness. Simple pitch discrimination thresholds were also found to be impaired by timbre changes that include brightness changes (Moore and Glasberg, 1990), even for musically-trained listeners (Borchert et al., 2011). In the latter two studies, the timbre changes consisted of changes in harmonic ranks, and therefore perceptual factors other than brightness could be responsible for the impairment. Using more subtle timbre differences that were more directly related to brightness, Singh and Hirsh (1992) and Warrier and Zatorre (2002) confirmed that the perception of small pitch differences was impaired by timbre changes. Interestingly, the effect was still observable but reduced when a tonal melodic context was presented before the pitch comparison (Warrier and Zatorre, 2002). Using a task requiring judgments on timbre rather than pitch, Marozeau and de Cheveigné (2007) observed a small but systematic effect of fundamental frequency on the perception of timbre for artificial sounds constructed to differ on the brightness dimension. The effect could be either viewed as showing interference of pitch and brightness, or as requiring a revision of the definition of brightness, to include a term related to the fundamental frequency (see also Handel and Erickson, 2004, for a related approach with natural timbre).

Finally, and perhaps particularly relevant for the present purposes, at least two studies investigated the possibility to convey tunes with sound sequences containing changes along dimensions other than pitch. Moore and Rosen (1979), mapping pitch differences to loudness differences, did not observe any tune recognition with loudness cues. In contrast, McDermott et al. (2008), using a similar paradigm, found that listeners were able to match pitch melodic contours with loudness contours and moreover found that brightness contours were also able to convey tunes.

The cause of the discrepancies between studies is not fully clear. Warrier and Zatorre (2002) reviewed the literature about pitch and timbre interactions and they suggested that contextual effects, such as the ones that they reported, may have had varying degrees of influence in the different studies. This idea is especially supported by the results of Krumhansl and Iverson (1992), who showed that pitch and timbre interact differently within isolated tones and within tone sequences. On the specific issue of sequences, an important difference between McDermott et al. (2008), who found no difference between pitch and loudness sequence processing, and Cousineau et al. (2009), who found a pitch-sequence processing advantage, is that discriminability was equated across dimensions for Cousineau et al. (2009) but not for McDermott et al. (2008). It is therefore an open question whether brightness sequences will be processed as efficiently as pitch sequences, once discriminability is factored out.

## Method

### Subjects

Six listeners (mean age = 23.3 years; SD = 2.2, 5 females) with no self-reported hearing disorder participated in the study. Listeners were not selected based on their musical abilities, as previous investigations showed that musical expertise was not correlated to sensitivity in the discrimination of pitch or loudness sequences (Cousineau et al., 2009). Listener’s musical training ranged from 0 to 18 years of formal training (mean = 9.66 years; SD = 6.53). All listeners provided written consent before their participation.

### Apparatus

The stimuli were generated with a sampling rate of 44.1 kHz and a resolution of 16 bits. They were delivered binaurally to the listener via an external soundcard (RME Fireface 800) and closed headphones (Beyerdynamic DT 770 Pro). The listener was seated in a double-walled sound-insulated booth (Eckel).

### Stimuli

**Figure 1:**
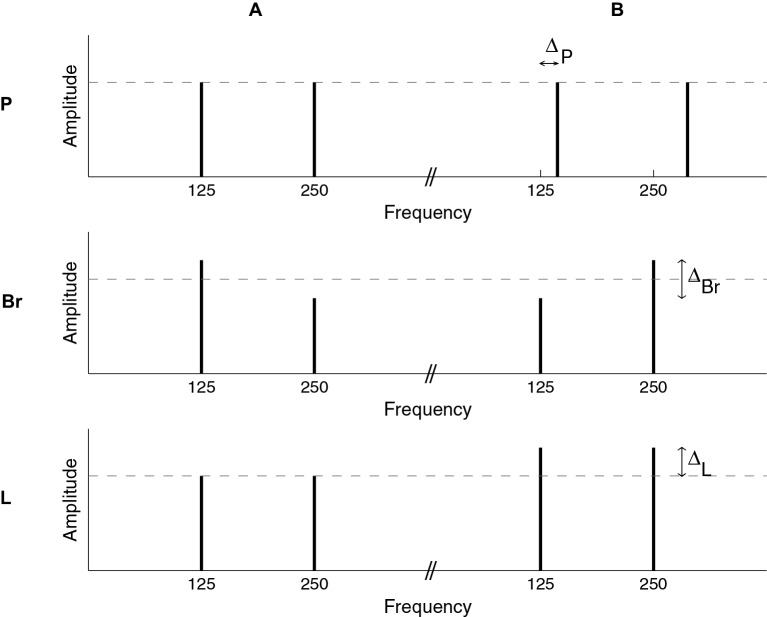
**Illustration of the stimuli used in the pitch (P), brightness (Br), and loudness (L) conditions.** The long-term spectra of the stimuli are schematized. For all conditions, individual sounds were two-tone complexes, with a one-octave frequency ratio. Sequences were constructed by the random succession of *A* and *B* complexes, which differed along a given dimension for each of the three conditions. In the P condition, the two components of each complex had the same amplitude (SPL); to create pitch sequences, *A* and *B* differed in terms of their fundamental frequency. In the Br condition, the two components of each complex had unequal amplitudes; the amplitude difference was varied between *A* and *B* to produce brightness sequences. In the L condition, the two components of each complex had the same amplitude, which was varied between *A* and *B* to create loudness sequences.

The building blocks for the sequences were complexes of two pure tones one octave apart (Figure 1). All tone complexes were 200 ms long. On and off raised-cosine ramps of 25 ms were applied.

There were three distinct stimulus conditions. In each condition, there was a reference stimulus, *A*, and another stimulus, *B*, differing from *A* by a fixed value, ∆, on the dimension of interest (Figure 1).

In the pitch^1^ condition (P), *A* had a fundamental frequency of 125 Hz while the fundamental frequency of *B* was 125 + ∆_P_ Hz. The two components of the complexes had a SPL of 71.5 dB, giving an overall SPL of 74.5 dB.

In the brightness condition (Br), *A* and *B* had a fundamental frequency of 125 Hz but the two components of the complexes had unequal amplitudes. In *A*, the lower-frequency tone was amplified by ∆_*Br*_/2 dB and the higher-frequency tone was attenuated by ∆_*Br*_/2 dB, all relative to a reference SPL of 71.5 dB. In *B*, the lower-frequency tone was attenuated and the higher-frequency tone was amplified, again by ∆_*Br*_/2 dB in both cases (see Figure 1). As a result, *B* was brighter than *A*.

In the loudness condition (L), *A* and *B* had a fundamental frequency of 125 Hz and the two components of each complex had equal amplitudes, but these amplitudes were not the same for *A* and *B*. The overall level was 75 dB for *A* and 75 + ∆_*L*_ dB for *B*.

### Adjustment task

A first part of the experiment aimed at choosing the ∆ values (∆_*P*_, ∆_*Br*_, and ∆_*L*_) to equate discriminability across all experimental conditions and for each listener. Details of the procedure can be found in Cousineau et al. (2009). Note that the specifics of this adjustment phase are not critical, as the adjustment itself was formally tested in the main part of the experiment (see below, conditions *N* = 1).

### Sequence discrimination task

In the subsequent and main part of the experiment, the ∆ values were used to construct sequences on each dimension of interest. Each element of a sequence was, at random, either stimulus *A* or stimulus *B*. The number of elements per sequence was experimentally varied, with values *N* = 1, 2, or 4. There was no silent gap between the elements of a sequence.

A constant-stimulus method was used to measure sequence discriminability. On each trial, a random sequence of *A*s and *B*s was first presented. After a 400 ms gap, a second sequence was presented. This second sequence could equiprobably be identical to the first sequence or different from it. In the latter case, a randomly selected element of the first sequence was changed, from *A* to *B* or vice versa. Listeners were asked whether the two sequences were the same or different. Keyboard presses were used to respond. No feedback was provided.

Four blocks of 50 trials were run for each listener, condition (P, Br, and L), and value of *N* (1, 2, and 4). Sensitivity was assessed with the index *d*′ of signal detection theory (MacMillan and Creelman, 2001), computed from the corresponding 200 trials per listener, condition, and *N*.

## Results

Results of the adjustment phase were as follows. In the P condition, we introduced on average a change of ∆_*P*_ = 0.41 semitones between *A* and *B* tones (standard deviation 0.12 st). In the Br condition, a change of ∆_*Br*_ = 1.07 dB (s.d. 0.43 dB) was used: from *A* to *B*, one of the tones was increased, and the other decreased, by ∆_*Br*_ (see Figure 1). Finally, in the L condition, the average change was ∆_*L*_ = 1.21 dB (s.d. 0.50 dB). It is possible to convert these values into a percentage of change relative to the base parameter, F0 for the P condition or linear SPL for the Br and L conditions. The ∆-values represented changes of 2.4, 28.1, and 32.2% for P, Br, and L, respectively.

**Figure 2:**
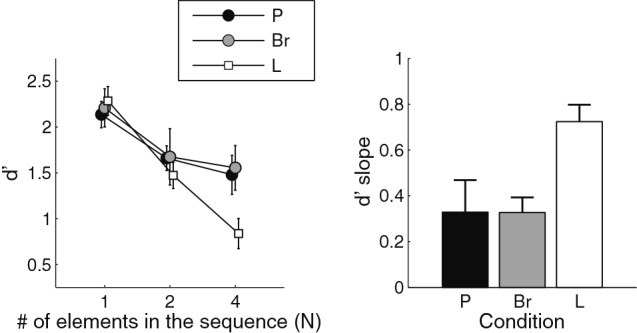
**Results for the sequence discrimination task.** *Left panel:* the mean and standard error about the mean for the sensitivity index *d*′ is presented, as a function of the number of elements in the sequences and the dimension along which the elements varied (P: pitch; Br: brightness; L: loudness). *Right panel:* the mean values and standard error about the mean for the *d*′-slope statistics. The *d*′-slope represents the drop in performance between *N* = 1 and *N* = 4 in the left panel. Smaller values indicate better sequence processing.

Results of the sequence discrimination task are displayed in Figure 2. The left panel shows the effect of *N* and condition on *d*′. Qualitatively, sensitivity was equivalent for all conditions when there was only one element per sequence (*N* = 1). In the L condition, sensitivity decreased rapidly when *N* increased. In the P condition, the drop in sensitivity was less pronounced. Both of those results were expected on the basis of Cousineau et al. (2009). In the Br condition, the pattern of results was similar to that obtained in the P condition, with only a modest effect of *N* on sensitivity.

The results were submitted to a repeated-measures analysis of variance (ANOVA; *N* × condition). The ANOVA revealed a main effect of *N* [*F*(2, 10) = 26.7, *p* < 0.0001, effect size generalized-*η*^2^ = 0.27], confirming that sensitivity decreased when sequence length increased. Crucially, a significant interaction between *N* and condition was also found [*F*(4, 20) = 4.26, *p* = 0.01, *η*^2^ = 0.06]. The decrease in sensitivity with *N* was therefore not the same for all conditions.

The sequence discrimination task at *N* = 1 can be viewed as a test of the adjustment phase, as the task then is equivalent to a classic same/different choice between two items. We ran post-hoc *t*-tests to compare sensitivity across the three conditions for *N* = 1. No significant difference was observed between conditions (Table 1). This shows that the adjustment phase had been successful on average: values of ∆_*P*_, ∆_*Br*_, and ∆_*L*_ were such that stimulus discriminability between *A*s and *B*s was indeed equated for all dimensions of interest.

**Table 1:** **Sensitivity d′ for *N* = 1 across conditions**.

**Comparison**	***t*(5)**	***p***
P-Br	0.251	0.8118
P-L	0.6763	0.3249
Br-L	0.5288	0.7584

*The d′ values for *N* = 1 were compared between conditions using two-sample paired t-tests. The table displays the outcome of the analysis (uncorrected) for all comparisons*.

In order to further characterize the change in sensitivity with increasing *N* in the three conditions, the amount of sensitivity change between *N* = 1 and *N* = 4 was estimated with the *d*′-slope statistic (Cousineau et al., 2009). For each condition, straight lines were fitted to the individual data points obtained for the different *N* values, using a log-scale for *N*. The slope of the fitted lines characterizes the effect of *N* on sensitivity, while normalizing for possible individual differences at *N* = 1. The outcome of this analysis is displayed in the right panel of Figure 2. We performed paired *t*-tests to compare the Br condition with the other two conditions. This showed that, with regard to *d*′-slope, the Br condition did not differ significantly from the P condition (*t*(5) = 2.49; *p* = 0.98), but was significantly different from the L condition (*t*(5) = 4.37; *p* = 0.007). Since the slopes were larger in the L condition, “long” sequences were less discriminable in that condition.

## Discussion

We used two-tone complexes to try and compare, in a simple situation, the perception of pitch, brightness, and loudness sequences. An adjustment phase equated discriminability between the elements of all sequences, irrespective of the perceptual dimension being manipulated. In accordance with previous results (Cousineau et al., 2009, 2010a,b), we found that pitch sequences were processed more “efficiently” than loudness sequences: the decrease in sensitivity with increasing number of sequence elements—from one to four—was less pronounced for pitch than for loudness. The novel result is that brightness sequences produced a pattern of sensitivity that was indistinguishable from the pattern obtained for pitch sequences, and markedly different from the pattern obtained for loudness sequences. This outcome is striking in view of the fact that physically, both the brightness and loudness sequences were based on manipulations of sound level, whereas the pitch sequences were based on manipulations of frequency.

What could be the reason for the strong similarity between pitch and brightness sequence processing? Obviously, even though we did not observe any significant difference nor any trend towards a difference, it is possible that more statistical power would distinguish between the two conditions. Note, however, that statistical power was sufficient to show a clear difference between brightness and loudness. Another possibility is that pitch and brightness sequences recruit completely distinct sensory processes, which nevertheless produce the same performance pattern in our paradigm. We do not rule out this possibility. Additional investigations using for instance adaptation paradigms (Shu et al., 1993) would be useful to further test this hypothesis.

Another, perhaps more parsimonious, explanation is that pitch and brightness share a common sequence-processing mechanism that is not available for processing loudness sequences. Previously, we hypothesized that the pitch-sequence advantage was due to frequency-shift detectors (FSDs, Cousineau et al., 2009). The existence of FSDs in the auditory system has been advocated, for instance, by Demany and Ramos (2005). They found that the direction of a frequency/pitch shift between two successive pure tones can be perceptually identified even when, in consequence of an informational masking effect, the pitch of the first tone cannot be heard out individually. This suggested that the direction of the frequency shift was encoded implicitly and independently from the explicit encoding of the individual tone frequencies, through FSDs. Findings similar to those of Demany and Ramos (2005) were reported by, e.g., Carcagno et al. (2011) and Moore et al. (2013). Additional behavioral evidence for FSDs can be found in adaptation paradigms (Okada and Kashino, 2003) or memory-capacity experiments (Demany et al., 2008). For our sequence task, as FSDs are expected to encode the relation between harmonics of successive tone complexes, listeners may have an additional source of information to process pitch sequences compared to loudness sequences. Also, because FSDs are thought to operate on individual frequency components, the advantage should be restricted to sequences that contain resolved harmonics. This is what is observed experimentally (Cousineau et al., 2010b).

Since we obtained here identical results for brightness sequences and pitch sequences, a conceivable hypothesis is that FSDs were recruited in the brightness sequence task and were able to encode brightness shifts. This would require an extension of the standard view of FSDs, which are supposed to react only to changes in the frequency of pure tones (e.g., Demany et al., 2009). In our brightness sequences, a louder tone at one of the two possible frequencies was followed by a softer tone at the same frequency, so there was no frequency shift for those two successive tones. A louder *high*-frequency tone was also followed by a louder *low*-frequency tone, and vice versa. If FSDs tracked the louder tones, they would see a frequency shift. Such a generalization of FSDs to any form of spectral shifts, including spectral envelope, may be consistent with behavioral data showing a contrastive after-effect following prolonged exposure to shifts in the spectral envelope of noise stimuli (Shu et al., 1993). However, at least for sounds with tonal components, previous research has suggested that the FSDs react optimally to small frequency shifts, around 1 or 1.5 semitone, and would be largely insensitive to shifts of one octave (Demany et al., 2009).

Another hypothesis is that FSDs were not the cause of efficient sequence processing, neither in the present experiment nor in the previous pitch-sequence experiments. One then has to consider a different mechanism, applicable to changes of pitch with resolved harmonics and to changes of brightness, but not available for changes of loudness or the pitch of unresolved harmonics. We do not have a specific proposal for what such a mechanism might be, but different possibilities can be envisioned. At the encoding stage, complex spectro-temporal processing such as observed in the mammalian auditory cortex (Klein et al., 2006) could be sensitive to both shifts in frequency at a narrow spectral scale and shifts in spectral center of gravity at broader spectral scale. A recent physiological study (Bizley et al., 2009) has indeed shown strong interactions, in the same neurons and over populations of neurons, for the encoding of pitch and timbre (vowel formants). At the memory stage, stores for pitch and brightness appear to be independent when tested with an interference paradigm (Semal and Demany, 1991; Starr and Pitt, 1997), but a common mechanism could be involved in the use and retrieval of the content of independent memory stores. Finally, pitch and brightness may both be able to recruit a common mechanism involved in relative (interval) coding. Some evidence has been found for the perception of timbre intervals (McAdams and Cunible, 1992), but without singling out the brightness dimension and with large inter-individual differences. McDermott et al. (2008) hypothesized a common central locus for the encoding of relative information for both pitch and brightness, to explain their common ability to convey musical tunes. However, the latter study also suggested that the same was true for loudness, whereas we consistently found poor performance with loudness sequences when discriminability was factored out. It must thus be acknowledged that, for now, the relationship between pitch and brightness sequence processing remains largely mysterious.

Before concluding, it is worth pointing out a few caveats of our experimental method. First, the tested listeners were not selected on the basis of their musical training and the effect of this factor on sequence processing could not be confidently assessed because the group was too small. As shown by Cousineau et al. (2009) with a larger group of listeners, musical training does not correlate with the efficiency of pitch-sequence or loudness-sequence processing. It remains conceivable, nevertheless, that musical training specifically affects *brightness*-sequence processing. However, this is highly unlikely since musicians are trained on pitch and not brightness sequences. Second, the acoustic parameter that was manipulated to induce brightness changes (the amplitude ratio between two octave-related component) is somewhat arbitrary. It was chosen as a simple way to modulate brightness, but it remains to be seen whether the results generalize to other brightness manipulations. As shown by previous investigations, the pitch-sequence advantage does not hold for all sounds that produce pitch; rather, only pitch sequences with resolved harmonics appear to be processed more efficiently than loudness sequences (Cousineau et al., 2009, 2010b). It would thus be of interest to investigate brightness sequences generated with, e.g., bandpass filtered complexes, resolved or unresolved. Third, it is tempting to put in parallel the efficiency of processing of brightness changes with the demands of speech processing, for which a number of meaningful cues are related to timbre (such as vowel formants or consonant formant transitions). But it is unclear to us whether the brightness changes investigated here are really representative of the timbre contrasts used in natural speech.

In conclusion, the present results can be considered in the light of the respective roles of pitch and brightness in music, as outlined in the introduction. If efficient perceptual processing is available for brightness sequences, why are they not used more often in music? Orthogonal to the issue of efficiency, there are several differences between the characteristics of pitch and brightness. For instance, brightness is coarser than pitch. Pitch also has a cyclic aspect due to octave similarity (Deutsch, 1982), whereas this is presumably not the case for brightness. Furthermore, for purely practical reasons, fine manipulations of brightness can be difficult to achieve with musical instruments (although counter examples are provided by the jaw harp, or the wah-wah pedal for the electric guitar). Yet another observation pertains to the effect of context, as it seems impossible to accurately process brightness changes in sequences where pitch also varies (Krumhansl and Iverson, 1992). So, even though the present results demonstrate that, perhaps surprisingly, pitch and brightness engage similarly efficient sequential processing in isolation, other factors may play a role in the musical uses of the two dimensions.

## Conflict of interest statement

The authors declare that the research was conducted in the absence of any commercial or financial relationships that could be construed as a potential conflict of interest.

## Footnotes

^1^For simplicity, we refer to each condition by the name of the perceptual dimension that was affected by a given acoustic manipulation. Each acoustic manipulation was unlikely to affect significantly perceptual dimensions other than the target dimension, but our results may not generalize to all possible acoustic manipulations affecting pitch, loudness or brightness (see Section Discussion).
